# Supplementary material for: Consumer Attitudes towards Food Preservation Methods
Source: Foods. 2022 May 6;11(9):1349. doi: 10.3390/foods11091349 (PMC9099755; doi:10.3390/foods11091349)
Supplement: Supplementary file 1 [file foods-11-01349-s001.zip › foods-1688306-supplementary.pdf]

Supplementary material

Table S1. Post Hoc Comparisons - Methods

		Mean Difference	SE	t	Cohen's d	p_{holm}
PRE	IRR	0.259	0.096	2.707	0.135	0.027
	RWV	-0.163	0.096	-1.702	-0.085	0.089
	MAP	-1.437	0.096	-15.007	-0.746	< .001
	STE	-2.072	0.096	-21.634	-1.075	< .001
	PAS	-2.741	0.096	-28.622	-1.422	< .001
	HPP	-1.817	0.096	-18.978	-0.943	< .001
	MWV	-0.373	0.096	-3.894	-0.193	< .001
IRR	RWV	-0.422	0.096	-4.409	-0.219	< .001
	MAP	-1.696	0.096	-17.714	-0.880	< .001
	STE	-2.331	0.096	-24.341	-1.210	< .001
	PAS	-3.000	0.096	-31.329	-1.557	< .001
	HPP	-2.077	0.096	-21.685	-1.078	< .001
	MWV	-0.632	0.096	-6.601	-0.328	< .001
RWV	MAP	-1.274	0.096	-13.305	-0.661	< .001
	STE	-1.909	0.096	-19.932	-0.990	< .001
	PAS	-2.578	0.096	-26.920	-1.338	< .001
	HPP	-1.654	0.096	-17.276	-0.858	< .001
	MWV	-0.210	0.096	-2.192	-0.109	0.057
MAP	STE	-0.635	0.096	-6.627	-0.329	< .001
	PAS	-1.304	0.096	-13.615	-0.677	< .001
	HPP	-0.380	0.096	-3.971	-0.197	< .001

Table S1. Post Hoc Comparisons - Methods

		Mean Difference	SE	t	Cohen's d	p holm
	MWV	1.064	0.096	11.113	0.552	< .001
STE	PAS	-0.669	0.096	-6.988	-0.347	< .001
	HPP	0.254	0.096	2.656	0.132	0.027
	MWV	1.699	0.096	17.740	0.882	< .001
PAS	HPP	0.923	0.096	9.644	0.479	< .001
	MWV	2.368	0.096	24.728	1.229	< .001
HPP	MWV	1.444	0.096	15.084	0.750	< .001

Note. Cohen's d does not correct for multiple comparisons.

Note. P-value adjusted for comparing a family of 28

Note. PRE – addition of preservatives; IRR – irradiation preservation; RWV – radio wave preservation; MAP – packaging in modified atmosphere preservation; STE – sterilisation; PAS – pasteurisation; HPP – high pressure processing; MWV – microwave preservation).

Table S2. Post Hoc Comparisons - In-store behaviour ✻ Methods

		Mean Difference	SE	t	p holm
No, Verify, PRE	Verify, PRE	0.014	0.163	0.087	1.000
	No, Verify, IRR	0.380	0.122	3.119	0.057
	Verify, IRR	0.079	0.163	0.482	1.000
	No, Verify, RWV	-0.008	0.122	-0.066	1.000
	Verify, RWV	-0.399	0.163	-2.439	0.400
	No, Verify, MAP	-1.292	0.122	-10.603	< .001
	Verify, MAP	-1.657	0.163	-10.136	< .001
	No, Verify, STE	-1.972	0.122	-16.184	< .001

Table S2. Post Hoc Comparisons - In-store behaviour ✻ Methods

		Mean Difference	SE	t	p_{holm}
	Verify, STE	-2.218	0.163	-13.570	< .001
	No, Verify, PAS	-2.732	0.122	-22.421	< .001
	Verify, PAS	-2.741	0.163	-16.767	< .001
	No, Verify, HPP	-1.712	0.122	-14.050	< .001
	Verify, HPP	-1.973	0.163	-12.070	< .001
	No, Verify, MWV	-0.260	0.122	-2.134	0.758
	Verify, MWV	-0.541	0.163	-3.308	0.033
Verify, PRE	No, Verify, IRR	0.366	0.163	2.238	0.633
	Verify, IRR	0.065	0.155	0.417	1.000
	No, Verify, RWV	-0.022	0.163	-0.136	1.000
	Verify, RWV	-0.413	0.155	-2.668	0.215
	No, Verify, MAP	-1.306	0.163	-7.991	< .001
	Verify, MAP	-1.671	0.155	-10.798	< .001
	No, Verify, STE	-1.986	0.163	-12.152	< .001
	Verify, STE	-2.232	0.155	-14.425	< .001
	No, Verify, PAS	-2.746	0.163	-16.801	< .001
	Verify, PAS	-2.755	0.155	-17.802	< .001
	No, Verify, HPP	-1.726	0.163	-10.561	< .001
	Verify, HPP	-1.987	0.155	-12.841	< .001
	No, Verify, MWV	-0.274	0.163	-1.678	1.000
	Verify, MWV	-0.555	0.155	-3.585	0.013
No, Verify, IRR	Verify, IRR	-0.301	0.163	-1.843	1.000

Table S2. Post Hoc Comparisons - In-store behaviour ✻ Methods

		Mean Difference	SE	t	p_{holm}
	No, Verify, RWV	-0.388	0.122	-3.184	0.048
	Verify, RWV	-0.779	0.163	-4.764	< .001
	No, Verify, MAP	-1.672	0.122	-13.722	< .001
	Verify, MAP	-2.037	0.163	-12.461	< .001
	No, Verify, STE	-2.352	0.122	-19.302	< .001
	Verify, STE	-2.598	0.163	-15.895	< .001
	No, Verify, PAS	-3.112	0.122	-25.539	< .001
	Verify, PAS	-3.121	0.163	-19.092	< .001
	No, Verify, HPP	-2.092	0.122	-17.168	< .001
	Verify, HPP	-2.353	0.163	-14.395	< .001
	No, Verify, MWV	-0.640	0.122	-5.252	< .001
	Verify, MWV	-0.921	0.163	-5.633	< .001
Verify, IRR	No, Verify, RWV	-0.087	0.163	-0.530	1.000
	Verify, RWV	-0.477	0.155	-3.085	0.060
	No, Verify, MAP	-1.371	0.163	-8.386	< .001
	Verify, MAP	-1.735	0.155	-11.215	< .001
	No, Verify, STE	-2.051	0.163	-12.546	< .001
	Verify, STE	-2.297	0.155	-14.842	< .001
	No, Verify, PAS	-2.811	0.163	-17.196	< .001
	Verify, PAS	-2.819	0.155	-18.219	< .001
	No, Verify, HPP	-1.791	0.163	-10.956	< .001
	Verify, HPP	-2.052	0.155	-13.257	< .001

Table S2. Post Hoc Comparisons - In-store behaviour ✻ Methods

		Mean Difference	SE	t	p_{holm}
	No, Verify, MWV	-0.339	0.163	-2.072	0.806
	Verify, MWV	-0.619	0.155	-4.002	0.003
No, Verify, RWV	Verify, RWV	-0.391	0.163	-2.390	0.440
	No, Verify, MAP	-1.284	0.122	-10.537	< .001
	Verify, MAP	-1.649	0.163	-10.087	< .001
	No, Verify, STE	-1.964	0.122	-16.118	< .001
	Verify, STE	-2.210	0.163	-13.521	< .001
	No, Verify, PAS	-2.724	0.122	-22.355	< .001
	Verify, PAS	-2.733	0.163	-16.718	< .001
	No, Verify, HPP	-1.704	0.122	-13.984	< .001
	Verify, HPP	-1.965	0.163	-12.021	< .001
	No, Verify, MWV	-0.252	0.122	-2.068	0.806
	Verify, MWV	-0.533	0.163	-3.259	0.039
Verify, RWV	No, Verify, MAP	-0.893	0.163	-5.465	< .001
	Verify, MAP	-1.258	0.155	-8.130	< .001
	No, Verify, STE	-1.573	0.163	-9.625	< .001
	Verify, STE	-1.819	0.155	-11.757	< .001
	No, Verify, PAS	-2.333	0.163	-14.275	< .001
	Verify, PAS	-2.342	0.155	-15.134	< .001
	No, Verify, HPP	-1.313	0.163	-8.035	< .001
	Verify, HPP	-1.574	0.155	-10.172	< .001
	No, Verify, MWV	0.139	0.163	0.849	1.000

Table S2. Post Hoc Comparisons - In-store behaviour ∗ Methods

		Mean Difference	SE	t	p_{holm}
	Verify, MWV	-0.142	0.155	-0.917	1.000
No, Verify, MAP	Verify, MAP	-0.365	0.163	-2.232	0.633
	No, Verify, STE	-0.680	0.122	-5.581	< .001
	Verify, STE	-0.926	0.163	-5.666	< .001
	No, Verify, PAS	-1.440	0.122	-11.818	< .001
	Verify, PAS	-1.449	0.163	-8.863	< .001
	No, Verify, HPP	-0.420	0.122	-3.447	0.021
	Verify, HPP	-0.681	0.163	-4.166	0.001
	No, Verify, MWV	1.032	0.122	8.469	< .001
	Verify, MWV	0.751	0.163	4.597	< .001
Verify, MAP	No, Verify, STE	-0.315	0.163	-1.929	0.971
	Verify, STE	-0.561	0.155	-3.627	0.011
	No, Verify, PAS	-1.075	0.163	-6.578	< .001
	Verify, PAS	-1.084	0.155	-7.004	< .001
	No, Verify, HPP	-0.055	0.163	-0.338	1.000
	Verify, HPP	-0.316	0.155	-2.043	0.806
	No, Verify, MWV	1.397	0.163	8.545	< .001
	Verify, MWV	1.116	0.155	7.212	< .001
No, Verify, STE	Verify, STE	-0.246	0.163	-1.505	1.000
	No, Verify, PAS	-0.760	0.122	-6.237	< .001
	Verify, PAS	-0.769	0.163	-4.703	< .001
	No, Verify, HPP	0.260	0.122	2.134	0.758

Table S2. Post Hoc Comparisons - In-store behaviour ∗ Methods

		Mean Difference	SE	t	p_{holm}
	Verify, HPP	-9.032e -4	0.163	-0.006	1.000
	No, Verify, MWV	1.712	0.122	14.050	< .001
	Verify, MWV	1.431	0.163	8.757	< .001
Verify, STE	No, Verify, PAS	-0.514	0.163	-3.144	0.054
	Verify, PAS	-0.523	0.155	-3.377	0.027
	No, Verify, HPP	0.506	0.163	3.096	0.060
	Verify, HPP	0.245	0.155	1.584	1.000
	No, Verify, MWV	1.958	0.163	11.979	< .001
	Verify, MWV	1.677	0.155	10.839	< .001
No, Verify, PAS	Verify, PAS	-0.009	0.163	-0.053	1.000
	No, Verify, HPP	1.020	0.122	8.371	< .001
	Verify, HPP	0.759	0.163	4.644	< .001
	No, Verify, MWV	2.472	0.122	20.287	< .001
	Verify, MWV	2.191	0.163	13.407	< .001
Verify, PAS	No, Verify, HPP	1.029	0.163	6.293	< .001
	Verify, HPP	0.768	0.155	4.961	< .001
	No, Verify, MWV	2.481	0.163	15.177	< .001
	Verify, MWV	2.200	0.155	14.216	< .001
No, Verify, HPP	Verify, HPP	-0.261	0.163	-1.596	1.000
	No, Verify, MWV	1.452	0.122	11.916	< .001
	Verify, MWV	1.171	0.163	7.166	< .001
Verify, HPP	No, Verify, MWV	1.713	0.163	10.480	< .001

Table S2. Post Hoc Comparisons - In-store behaviour ✻ Methods

		Mean Difference	SE	t	p_{holm}
	Verify, MWV	1.432	0.155	9.255	< .001
No, Verify, MWV	Verify, MWV	-0.281	0.163	-1.717	1.000

Note. P-value adjusted for comparing a family of 120
Note. PRE – addition of preservatives; IRR – irradiation preservation; RWV – radio wave preservation; MAP – packaging in modified atmosphere preservation; STE – sterilisation; PAS – pasteurisation; HPP – high pressure processing; MWV – microwave preservation).

Table S3. Post Hoc Comparisons - Education level ✻ Methods

		Mean Difference	SE	t	p_{holm}
1, PRE	2, PRE	-0.423	0.195	-2.170	1.000
	3, PRE	0.280	0.195	1.439	1.000
	1, IRR	0.395	0.168	2.348	1.000
	2, IRR	0.172	0.195	0.881	1.000
	3, IRR	0.077	0.195	0.397	1.000
	1, RWV	0.023	0.168	0.138	1.000
	2, RWV	-0.292	0.195	-1.501	1.000
	3, RWV	-0.350	0.195	-1.798	1.000
	1, MAP	-1.132	0.168	-6.722	< .001
	2, MAP	-1.510	0.195	-7.752	< .001
	3, MAP	-1.792	0.195	-9.204	< .001
	1, STE	-1.915	0.168	-11.372	< .001

Table S3. Post Hoc Comparisons - Education level ✻ Methods

		Mean Difference	SE	t	p_{holm}
	2, STE	-2.162	0.195	-11.101	< .001
	3, STE	-2.270	0.195	-11.660	< .001
	1, PAS	-2.984	0.168	-17.725	< .001
	2, PAS	-2.495	0.195	-12.813	< .001
	3, PAS	-2.901	0.195	-14.897	< .001
	1, HPP	-1.504	0.168	-8.932	< .001
	2, HPP	-2.104	0.195	-10.804	< .001
	3, HPP	-1.966	0.195	-10.097	< .001
	1, MWV	-0.016	0.168	-0.092	1.000
	2, MWV	-0.589	0.195	-3.026	0.205
	3, MWV	-0.633	0.195	-3.250	0.101
2, PRE	3, PRE	0.703	0.191	3.672	0.024
	1, IRR	0.818	0.195	4.201	0.003
	2, IRR	0.594	0.163	3.650	0.025
	3, IRR	0.500	0.191	2.612	0.653
	1, RWV	0.446	0.195	2.290	1.000
	2, RWV	0.130	0.163	0.801	1.000
	3, RWV	0.072	0.191	0.379	1.000
	1, MAP	-0.709	0.195	-3.642	0.026
	2, MAP	-1.087	0.163	-6.677	< .001
	3, MAP	-1.370	0.191	-7.155	< .001
	1, STE	-1.492	0.195	-7.662	< .001

Table S3. Post Hoc Comparisons - Education level ✻ Methods

		Mean Difference	SE	t	p_{holm}
	2, STE	-1.739	0.163	-10.683	< .001
	3, STE	-1.848	0.191	-9.653	< .001
	1, PAS	-2.562	0.195	-13.156	< .001
	2, PAS	-2.072	0.163	-12.731	< .001
	3, PAS	-2.478	0.191	-12.947	< .001
	1, HPP	-1.081	0.195	-5.552	< .001
	2, HPP	-1.681	0.163	-10.327	< .001
	3, HPP	-1.543	0.191	-8.063	< .001
	1, MWV	0.407	0.195	2.091	1.000
	2, MWV	-0.167	0.163	-1.024	1.000
	3, MWV	-0.210	0.191	-1.098	1.000
3, PRE	1, IRR	0.115	0.195	0.591	1.000
	2, IRR	-0.109	0.191	-0.568	1.000
	3, IRR	-0.203	0.163	-1.246	1.000
	1, RWV	-0.257	0.195	-1.320	1.000
	2, RWV	-0.572	0.191	-2.991	0.228
	3, RWV	-0.630	0.163	-3.873	0.011
	1, MAP	-1.412	0.195	-7.251	< .001
	2, MAP	-1.790	0.191	-9.350	< .001
	3, MAP	-2.072	0.163	-12.731	< .001
	1, STE	-2.195	0.195	-11.272	< .001
	2, STE	-2.442	0.191	-12.758	< .001

Table S3. Post Hoc Comparisons - Education level ✻ Methods

		Mean Difference	SE	t	p_{holm}
	3, STE	-2.551	0.163	-15.669	< .001
	1, PAS	-3.265	0.195	-16.766	< .001
	2, PAS	-2.775	0.191	-14.499	< .001
	3, PAS	-3.181	0.163	-19.541	< .001
	1, HPP	-1.784	0.195	-9.162	< .001
	2, HPP	-2.384	0.191	-12.455	< .001
	3, HPP	-2.246	0.163	-13.799	< .001
	1, MWV	-0.296	0.195	-1.519	1.000
	2, MWV	-0.870	0.191	-4.543	< .001
	3, MWV	-0.913	0.163	-5.609	< .001
1, IRR	2, IRR	-0.224	0.195	-1.149	1.000
	3, IRR	-0.318	0.195	-1.633	1.000
	1, RWV	-0.372	0.168	-2.210	1.000
	2, RWV	-0.688	0.195	-3.531	0.039
	3, RWV	-0.746	0.195	-3.829	0.013
	1, MAP	-1.527	0.168	-9.070	< .001
	2, MAP	-1.905	0.195	-9.783	< .001
	3, MAP	-2.188	0.195	-11.234	< .001
	1, STE	-2.310	0.168	-13.720	< .001
	2, STE	-2.557	0.195	-13.132	< .001
	3, STE	-2.666	0.195	-13.690	< .001
	1, PAS	-3.380	0.168	-20.073	< .001

Table S3. Post Hoc Comparisons - Education level ✻ Methods

		Mean Difference	SE	t	p_{holm}
	2, PAS	-2.890	0.195	-14.844	< .001
	3, PAS	-3.296	0.195	-16.927	< .001
	1, HPP	-1.899	0.168	-11.280	< .001
	2, HPP	-2.499	0.195	-12.834	< .001
	3, HPP	-2.361	0.195	-12.127	< .001
	1, MWV	-0.411	0.168	-2.440	1.000
	2, MWV	-0.985	0.195	-5.057	< .001
	3, MWV	-1.028	0.195	-5.280	< .001
2, IRR	3, IRR	-0.094	0.191	-0.492	1.000
	1, RWV	-0.148	0.195	-0.762	1.000
	2, RWV	-0.464	0.163	-2.849	0.340
	3, RWV	-0.522	0.191	-2.726	0.479
	1, MAP	-1.303	0.195	-6.693	< .001
	2, MAP	-1.681	0.163	-10.327	< .001
	3, MAP	-1.964	0.191	-10.259	< .001
	1, STE	-2.086	0.195	-10.714	< .001
	2, STE	-2.333	0.163	-14.333	< .001
	3, STE	-2.442	0.191	-12.758	< .001
	1, PAS	-3.156	0.195	-16.207	< .001
	2, PAS	-2.667	0.163	-16.381	< .001
	3, PAS	-3.072	0.191	-16.051	< .001
	1, HPP	-1.675	0.195	-8.604	< .001

Table S3. Post Hoc Comparisons - Education level ✻ Methods

		Mean Difference	SE	t	p holm
	2, HPP	-2.275	0.163	-13.977	< .001
	3, HPP	-2.138	0.191	-11.168	< .001
	1, MWV	-0.187	0.195	-0.961	1.000
	2, MWV	-0.761	0.163	-4.674	< .001
	3, MWV	-0.804	0.191	-4.202	0.003
3, IRR	1, RWV	-0.054	0.195	-0.278	1.000
	2, RWV	-0.370	0.191	-1.931	1.000
	3, RWV	-0.428	0.163	-2.626	0.634
	1, MAP	-1.209	0.195	-6.209	< .001
	2, MAP	-1.587	0.191	-8.291	< .001
	3, MAP	-1.870	0.163	-11.484	< .001
	1, STE	-1.992	0.195	-10.230	< .001
	2, STE	-2.239	0.191	-11.698	< .001
	3, STE	-2.348	0.163	-14.422	< .001
	1, PAS	-3.062	0.195	-15.724	< .001
	2, PAS	-2.572	0.191	-13.439	< .001
	3, PAS	-2.978	0.163	-18.295	< .001
	1, HPP	-1.581	0.195	-8.120	< .001
	2, HPP	-2.181	0.191	-11.395	< .001
	3, HPP	-2.043	0.163	-12.553	< .001
	1, MWV	-0.093	0.195	-0.477	1.000
	2, MWV	-0.667	0.191	-3.483	0.046

Table S3. Post Hoc Comparisons - Education level ∗ Methods

		Mean Difference	SE	t	p_{holm}
	3, MWV	-0.710	0.163	-4.362	0.001
1, RWV	2, RWV	-0.315	0.195	-1.620	1.000
	3, RWV	-0.373	0.195	-1.918	1.000
	1, MAP	-1.155	0.168	-6.860	< .001
	2, MAP	-1.533	0.195	-7.872	< .001
	3, MAP	-1.815	0.195	-9.323	< .001
	1, STE	-1.938	0.168	-11.510	< .001
	2, STE	-2.185	0.195	-11.221	< .001
	3, STE	-2.294	0.195	-11.779	< .001
	1, PAS	-3.008	0.168	-17.863	< .001
	2, PAS	-2.518	0.195	-12.933	< .001
	3, PAS	-2.924	0.195	-15.017	< .001
	1, HPP	-1.527	0.168	-9.070	< .001
	2, HPP	-2.127	0.195	-10.923	< .001
	3, HPP	-1.989	0.195	-10.216	< .001
	1, MWV	-0.039	0.168	-0.230	1.000
	2, MWV	-0.613	0.195	-3.146	0.141
	3, MWV	-0.656	0.195	-3.369	0.067
2, RWV	3, RWV	-0.058	0.191	-0.303	1.000
	1, MAP	-0.840	0.195	-4.311	0.002
	2, MAP	-1.217	0.163	-7.478	< .001
	3, MAP	-1.500	0.191	-7.836	< .001

Table S3. Post Hoc Comparisons - Education level ∗ Methods

		Mean Difference	SE	t	p_{holm}
	1, STE	-1.623	0.195	-8.332	< .001
	2, STE	-1.870	0.163	-11.484	< .001
	3, STE	-1.978	0.191	-10.335	< .001
	1, PAS	-2.692	0.195	-13.826	< .001
	2, PAS	-2.203	0.163	-13.532	< .001
	3, PAS	-2.609	0.191	-13.628	< .001
	1, HPP	-1.212	0.195	-6.222	< .001
	2, HPP	-1.812	0.163	-11.128	< .001
	3, HPP	-1.674	0.191	-8.745	< .001
	1, MWV	0.277	0.195	1.421	1.000
	2, MWV	-0.297	0.163	-1.825	1.000
	3, MWV	-0.341	0.191	-1.779	1.000
3, RWV	1, MAP	-0.782	0.195	-4.014	0.006
	2, MAP	-1.159	0.191	-6.057	< .001
	3, MAP	-1.442	0.163	-8.858	< .001
	1, STE	-1.565	0.195	-8.034	< .001
	2, STE	-1.812	0.191	-9.464	< .001
	3, STE	-1.920	0.163	-11.796	< .001
	1, PAS	-2.634	0.195	-13.528	< .001
	2, PAS	-2.145	0.191	-11.205	< .001
	3, PAS	-2.551	0.163	-15.669	< .001
	1, HPP	-1.154	0.195	-5.925	< .001

Table S3. Post Hoc Comparisons - Education level ✻ Methods

		Mean Difference	SE	t	p_{holm}
	2, HPP	-1.754	0.191	-9.161	< .001
	3, HPP	-1.616	0.163	-9.926	< .001
	1, MWV	0.335	0.195	1.719	1.000
	2, MWV	-0.239	0.191	-1.249	1.000
	3, MWV	-0.283	0.163	-1.736	1.000
1, MAP	2, MAP	-0.378	0.195	-1.940	1.000
	3, MAP	-0.660	0.195	-3.392	0.063
	1, STE	-0.783	0.168	-4.650	< .001
	2, STE	-1.030	0.195	-5.289	< .001
	3, STE	-1.139	0.195	-5.848	< .001
	1, PAS	-1.853	0.168	-11.004	< .001
	2, PAS	-1.363	0.195	-7.001	< .001
	3, PAS	-1.769	0.195	-9.085	< .001
	1, HPP	-0.372	0.168	-2.210	1.000
	2, HPP	-0.972	0.195	-4.992	< .001
	3, HPP	-0.834	0.195	-4.285	0.002
	1, MWV	1.116	0.168	6.630	< .001
	2, MWV	0.542	0.195	2.786	0.410
	3, MWV	0.499	0.195	2.562	0.743
2, MAP	3, MAP	-0.283	0.191	-1.476	1.000
	1, STE	-0.405	0.195	-2.080	1.000
	2, STE	-0.652	0.163	-4.006	0.007

Table S3. Post Hoc Comparisons - Education level ∗ Methods

		Mean Difference	SE	t	p holm
	3, STE	-0.761	0.191	-3.975	0.007
	1, PAS	-1.475	0.195	-7.574	< .001
	2, PAS	-0.986	0.163	-6.054	< .001
	3, PAS	-1.391	0.191	-7.268	< .001
	1, HPP	0.006	0.195	0.029	1.000
	2, HPP	-0.594	0.163	-3.650	0.025
	3, HPP	-0.457	0.191	-2.385	1.000
	1, MWV	1.494	0.195	7.673	< .001
	2, MWV	0.920	0.163	5.653	< .001
	3, MWV	0.877	0.191	4.581	< .001
3, MAP	1, STE	-0.123	0.195	-0.629	1.000
	2, STE	-0.370	0.191	-1.931	1.000
	3, STE	-0.478	0.163	-2.938	0.260
	1, PAS	-1.192	0.195	-6.123	< .001
	2, PAS	-0.703	0.191	-3.672	0.024
	3, PAS	-1.109	0.163	-6.811	< .001
	1, HPP	0.288	0.195	1.481	1.000
	2, HPP	-0.312	0.191	-1.628	1.000
	3, HPP	-0.174	0.163	-1.068	1.000
	1, MWV	1.777	0.195	9.124	< .001
	2, MWV	1.203	0.191	6.284	< .001
	3, MWV	1.159	0.163	7.122	< .001

Table S3. Post Hoc Comparisons - Education level ✻ Methods

		Mean Difference	SE	t	p_{holm}
1, STE	2, STE	-0.247	0.195	-1.269	1.000
	3, STE	-0.356	0.195	-1.827	1.000
	1, PAS	-1.070	0.168	-6.353	< .001
	2, PAS	-0.580	0.195	-2.980	0.233
	3, PAS	-0.986	0.195	-5.064	< .001
	1, HPP	0.411	0.168	2.440	1.000
	2, HPP	-0.189	0.195	-0.971	1.000
	3, HPP	-0.051	0.195	-0.264	1.000
	1, MWV	1.899	0.168	11.280	< .001
	2, MWV	1.325	0.195	6.806	< .001
	3, MWV	1.282	0.195	6.583	< .001
2, STE	3, STE	-0.109	0.191	-0.568	1.000
	1, PAS	-0.823	0.195	-4.225	0.003
	2, PAS	-0.333	0.163	-2.048	1.000
	3, PAS	-0.739	0.191	-3.861	0.012
	1, HPP	0.658	0.195	3.379	0.065
	2, HPP	0.058	0.163	0.356	1.000
	3, HPP	0.196	0.191	1.022	1.000
	1, MWV	2.146	0.195	11.022	< .001
	2, MWV	1.572	0.163	9.659	< .001
	3, MWV	1.529	0.191	7.988	< .001
3, STE	1, PAS	-0.714	0.195	-3.667	0.024

Table S3. Post Hoc Comparisons - Education level ✻ Methods

		Mean Difference	SE	t	p_{holm}
	2, PAS	-0.225	0.191	-1.174	1.000
	3, PAS	-0.630	0.163	-3.873	0.011
	1, HPP	0.767	0.195	3.937	0.009
	2, HPP	0.167	0.191	0.871	1.000
	3, HPP	0.304	0.163	1.870	1.000
	1, MWV	2.255	0.195	11.580	< .001
	2, MWV	1.681	0.191	8.783	< .001
	3, MWV	1.638	0.163	10.060	< .001
1, PAS	2, PAS	0.489	0.195	2.513	0.843
	3, PAS	0.084	0.195	0.429	1.000
	1, HPP	1.481	0.168	8.794	< .001
	2, HPP	0.881	0.195	4.523	< .001
	3, HPP	1.018	0.195	5.230	< .001
	1, MWV	2.969	0.168	17.633	< .001
	2, MWV	2.395	0.195	12.300	< .001
	3, MWV	2.352	0.195	12.077	< .001
2, PAS	3, PAS	-0.406	0.191	-2.120	1.000
	1, HPP	0.991	0.195	5.090	< .001
	2, HPP	0.391	0.163	2.404	1.000
	3, HPP	0.529	0.191	2.764	0.433
	1, MWV	2.480	0.195	12.734	< .001
	2, MWV	1.906	0.163	11.707	< .001

Table S3. Post Hoc Comparisons - Education level ✻ Methods

		Mean Difference	SE	t	p_{holm}
	3, MWV	1.862	0.191	9.729	< .001
3, PAS	1, HPP	1.397	0.195	7.174	< .001
	2, HPP	0.797	0.191	4.164	0.003
	3, HPP	0.935	0.163	5.742	< .001
	1, MWV	2.885	0.195	14.818	< .001
	2, MWV	2.312	0.191	12.076	< .001
	3, MWV	2.268	0.163	13.933	< .001
1, HPP	2, HPP	-0.600	0.195	-3.081	0.174
	3, HPP	-0.462	0.195	-2.374	1.000
	1, MWV	1.488	0.168	8.840	< .001
	2, MWV	0.915	0.195	4.697	< .001
	3, MWV	0.871	0.195	4.473	< .001
2, HPP	3, HPP	0.138	0.191	0.719	1.000
	1, MWV	2.088	0.195	10.724	< .001
	2, MWV	1.514	0.163	9.303	< .001
	3, MWV	1.471	0.191	7.685	< .001
3, HPP	1, MWV	1.951	0.195	10.017	< .001
	2, MWV	1.377	0.191	7.193	< .001
	3, MWV	1.333	0.163	8.190	< .001
1, MWV	2, MWV	-0.574	0.195	-2.947	0.257
	3, MWV	-0.617	0.195	-3.170	0.131
2, MWV	3, MWV	-0.043	0.191	-0.227	1.000

Table S3. Post Hoc Comparisons - Education level ✻ Methods

	Mean Difference	SE	t	p holm

Note. P-value adjusted for comparing a family of 276

Note. PRE – addition of preservatives; IRR – irradiation preservation; RWV – radio wave preservation; MAP – packaging in modified atmosphere preservation; STE – sterilisation; PAS – pasteurisation; HPP – high pressure processing; MWV – microwave preservation).
